# Elucidating the Neuropathologic Mechanisms of SARS-CoV-2 Infection

**DOI:** 10.3389/fneur.2021.660087

**Published:** 2021-04-12

**Authors:** Mar Pacheco-Herrero, Luis O. Soto-Rojas, Charles R. Harrington, Yazmin M. Flores-Martinez, Marcos M. Villegas-Rojas, Alfredo M. León-Aguilar, Paola A. Martínez-Gómez, B. Berenice Campa-Córdoba, Ricardo Apátiga-Pérez, Carolin N. Corniel-Taveras, Jesabelle de J. Dominguez-García, Víctor Manuel Blanco-Alvarez, José Luna-Muñoz

**Affiliations:** ^1^Neuroscience Research Laboratory, Faculty of Health Sciences, Pontificia Universidad Católica Madre y Maestra, Santiago de los Caballeros, Dominican Republic; ^2^Facultad de Estudios Superiores Iztacala, Universidad Nacional Autónoma de México, Mexico City, Mexico; ^3^School of Medicine, Medical Sciences and Nutrition, University of Aberdeen, Aberdeen, United Kingdom; ^4^Programa Institucional de Biomedicina Molecular, Escuela Nacional de Medicina y Homeopatía, Instituto Politécnico Nacional, Mexico City, Mexico; ^5^Unidad Profesional Interdisciplinaria de Biotecnología del Instituto Politécnico Nacional (UPIBI- IPN), Mexico City, Mexico; ^6^Departamento de Fisiología, Escuela Nacional de Ciencias Biológicas, Instituto Politécnico Nacional, Mexico City, Mexico; ^7^National Dementia BioBank, Ciencias Biológicas, Facultad de Estudios Superiores, Cuautitlán, Mexico; ^8^Facultad de Enfermeria, Benemérita Universidad Autónoma de Puebla, Puebla, Mexico; ^9^Banco Estado de Cerebros-UNPHU, Universidad Nacional Pedro Henriquez Ureña, Santo Domingo, Dominican Republic

**Keywords:** SARS-CoV-2, storm cytokine syndrome, neuroinflammation, blood-brain barrier, neurological alterations, neurodegenerative diseases, Alzheimer's disease

## Abstract

The current pandemic caused by the new severe acute respiratory syndrome coronavirus 2 (SARS-CoV-2) has become a public health emergency. To date, March 1, 2021, coronavirus disease 2019 (COVID-19) has caused about 114 million accumulated cases and 2.53 million deaths worldwide. Previous pieces of evidence suggest that SARS-CoV-2 may affect the central nervous system (CNS) and cause neurological symptoms in COVID-19 patients. It is also known that angiotensin-converting enzyme-2 (ACE2), the primary receptor for SARS-CoV-2 infection, is expressed in different brain areas and cell types. Thus, it is hypothesized that infection by this virus could generate or exacerbate neuropathological alterations. However, the molecular mechanisms that link COVID-19 disease and nerve damage are unclear. In this review, we describe the routes of SARS-CoV-2 invasion into the central nervous system. We also analyze the neuropathologic mechanisms underlying this viral infection, and their potential relationship with the neurological manifestations described in patients with COVID-19, and the appearance or exacerbation of some neurodegenerative diseases.

## Introduction

Coronavirus disease 2019 (COVID-19) was first reported in December 2019 in Wuhan, in Hubei province, China (1). In March 2020, the world health organization (WHO) declared the COVID-19 a pandemic. Almost a year later, on March 1, 2021, more than 114 million cases and 2.53 million deaths have been reported (2–4). Severe acute respiratory syndrome coronavirus 2 (SARS-CoV-2), the causative agent of COVID-19, is 100 nm with an oval shape and covered with crown-shaped glycoprotein spikes (5). It is transmitted through respiratory droplets from infected individuals or contact with fomites. Once SARS-CoV-2 enters the body, the onset of symptoms ranges from 2 to 14 days. Patients can manifest clinically from asymptomatic or mild symptoms to moderate or severe symptoms (6, 7). Mild to moderate symptoms manifest as fever, dry cough, nasal congestion, sore throat, runny nose, fatigue, myalgias, diarrhea, anosmia, and ageusia as main symptoms (6, 7). The severe condition is characterized by atypical pneumonia, which can be observed as “ground-glass opacification” with bilateral multi-lobular consolidations by imaging studies (8). Between 5 and 30% of patients develop acute respiratory distress syndrome, characterized by rapid onset and with generalized inflammation in the lungs, requiring invasive life support therapy, such as mechanical ventilation (6, 7). There is increasing evidence that these critically ill COVID-19 patients suffer a so-called “cytokine storm syndrome,” characterized by the release of many pro-inflammatory cytokines [interleukin (IL)-1β and IL-6] and a low number of T cells into the bloodstream (9). The mean period from the onset of the symptoms to death is around 13 days [interquartile range (IQR) 11–18 days], and this depends on advanced age (>65 years) and comorbidities such as diabetes mellitus (DM), hypertension, cardiovascular disease, or chronic obstructive pulmonary disease (COPD) (10). These comorbidities are also risk factors for severity and transfer to intensive care unit (ICU), endotracheal intubation, and death in patients with COVID-19 (11). However, there may be bias in the epidemiological data due to the in-hospital stay of the patient, as well as the human development index of each country. SARS-CoV-2 also may be able to invade multiple organs, including the nervous system, and thus cause multiple organ dysfunction syndrome (MODS) (12). The neurological manifestations are beginning to take on unquestionable importance, mainly in the critical patient (13, 14). Neurological manifestations of COVID-19 and other coronavirus infections involve febrile seizures, disorientation, difficulty in speaking, encephalitis, and stroke (15–18). The mechanisms by which SARS-CoV-2 can spread, infect, cause damage to nerve cells and finally affect both the central (CNS) and peripheral (PNS) nervous system, are not yet understood. This review will analyze the potential mechanisms by which SARS-CoV-2 can invade the CNS and PNS and generate a neurotoxic environment that may trigger or worsen neurological disorders.

### SARS-CoV-2: Structure and Mechanism of Infection

**Figure 1:**
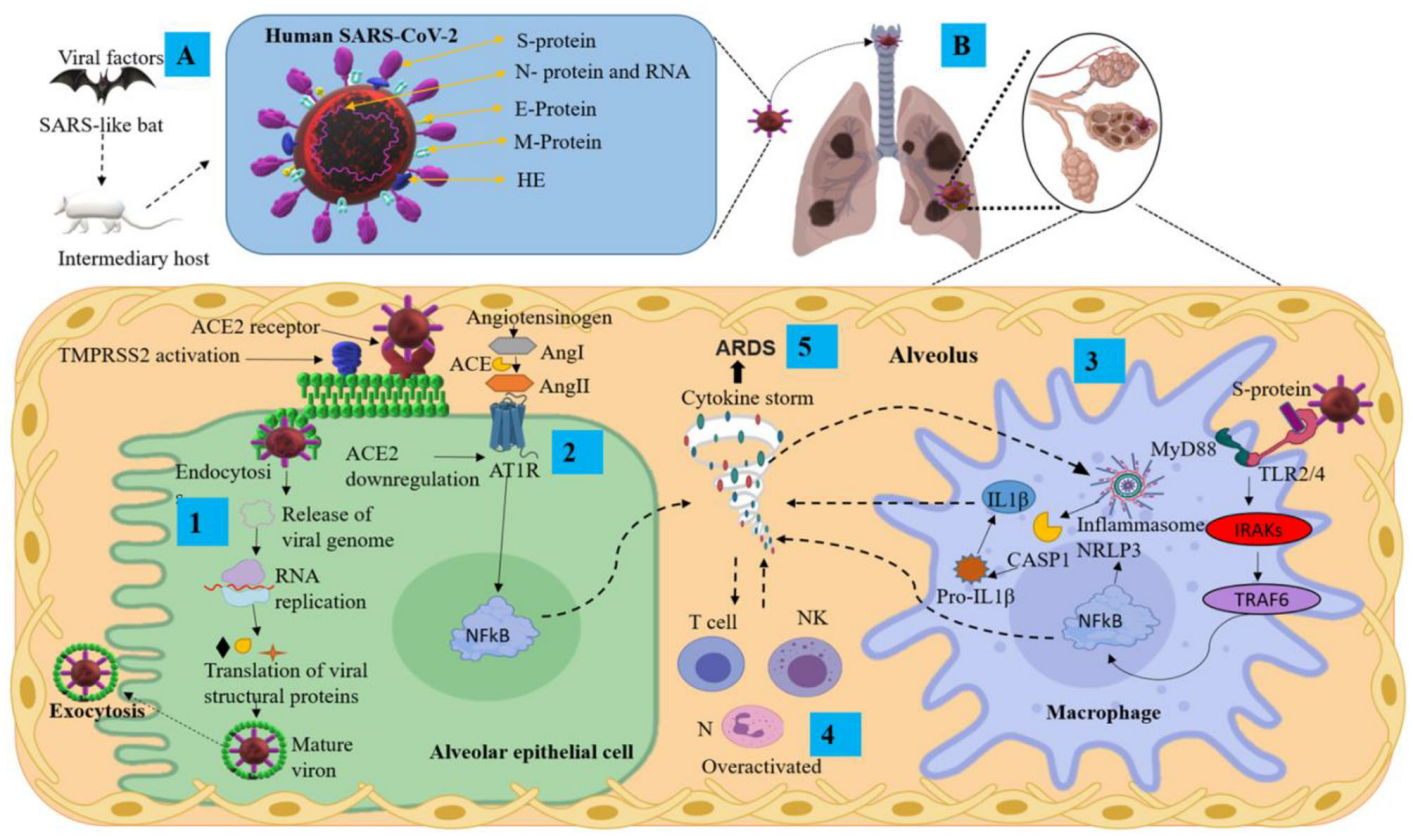
Pathological mechanisms of SARS-CoV-2 in the pulmonary alveolus. **(A)** Mode of transmission and main structural proteins of SARS-CoV-2. **(B)** Mechanisms of SARS-CoV-2 infection and pulmonary inflammatory immune response. ACE2, angiotensin-converting enzyme 2; Ang, angiotensin; ARDS, acute respiratory distress syndrome; AT1R, angiotensin II type I receptor; CASP1, aaspase 1; E protein, envelope small membrane protein; HE, hemagglutinin esterase; IL1β, interleukin 1 beta; IRAKs, interleukin-1 receptor-associated kinases; M protein, membrane protein; MyD88, myeloid differentiation primary response 88; N protein, nucleoprotein; N, neutrophils; NF-κB, nuclear factor Kappa B; NK, natural killer cells; NLRP3, nucleotide-binding domain-, leucine-rich repeat-containing receptor, pyrin domain-containing 3; RNA, ribonucleic acid; S protein, spike protein; TMPRSS2, transmembrane serine protease 2; TRAF6, tumor necrosis factor receptor-associated factor 6.

The coronaviruses (CoVs) belong to the *Orthocoronaviridae* subfamily; order: *Nidovirales*; subordination: *Cornidovirineae*; family: Coronaviridae (19). They can be grouped into four genera, including α/β/γ/δ-CoV: α and β infect mammals and γ/δ infect birds (20). CoVs are large, positive-stranded RNA viruses, and they are enveloped with a lipid membrane derived from a host cell. The protein protruding from the virus membrane is the spike (S) protein, giving the virus the appearance of a solar corona (1) (Figure 1A). Coronaviruses have single-stranded RNA of between 26.4 and 31.7 kilobases, making them the largest of RNA viruses (21). CoVs have several main structural proteins (Figure 1A): nucleocapsid (N) proteins, which surround the RNA genome; membrane (M) proteins (also known as E1 membrane glycoprotein or matrix protein) (20); envelope (E) proteins, involved in virus assembly, and S protein, which mediates virus entry into host cells. Some CoVs also encode an envelope-associated hemaglutinin-esterase protein (HE) used as an invading mechanism (22).

The S protein is the main antigenic component of SARS-CoV-2 structural proteins and is comprised of two subunits, S1 and S2 (23). This protein is multifunctional, contributing to host receptor binding, pathogenesis, and cell tropism. The S protein binds to host receptors on target cells, inducing virion particle endocytosis, and then catalyzes the fusion between host and viral membranes, allowing the virus genome penetration into the host cytoplasm (24). The S1 domain has a high-affinity association with the host receptor angiotensin-converting enzyme 2 (ACE2) (25). The receptor-binding domain (RBD) of the S protein binds to the extracellular peptidase domain of ACE2, mediating cell entry (25, 26). SARS-CoV-2 uses the SARS-CoV receptor ACE2 for entry and the transmembrane serine protease 2 (TMPRSS2) for S protein priming. The endosomal cysteine proteases cathepsin B and L (CatB/L) can be used to mature the S protein (27, 28). However, while TMPRSS2 is indispensable for viral spread and pathogenesis, the CatB/L activity is not essential (Figure 1B step 1). Once the virus enters the host cell, viral replication begins with translation of the replicase-polymerase gene and assembly of the replication-transcription complex. This complex also transcribes the genomic regions to structural proteins. New virions are assembled in the endoplasmic reticulum and Golgi apparatus released from the cell (Figure 1B step 1) (29). Finally, the newly assembled SARS-CoV-2 virions possess protein S on the surface and are ready to infect any cell that expresses the ACE2 receptor with no further requirement for TMPRSS2 activity (30).

### SARS-CoV2 Pathophysiology

The SARS-CoV-2 pathophysiology is not yet clear. It has been suggested that it can be similar to SARS-CoV (31, 32) with two possible responses (32):

1) After the viral infection occurs, active viral replication and dissemination through ACE2 receptors occurs with the associated host antiviral responses. SARS-CoV-2 downregulates ACE2 receptors, with loss of their catalytic effect at the membrane surface. Inflammation and thrombosis have been related to enhanced and unimpeded angiotensin II effects through the ACE-Angiotensin II-AT1 receptor axis (33) (Figure 1B step 2). The SARS-CoV-2 infection can lead to an acute immune response. This response is driven by inflammatory alveolar and monocyte-derived macrophages that can be activated by pathogen-associated molecular patterns (PAMPs) and damage-associated molecular patterns (DAMPs) released by infected pneumocytes (34–36). Subsequently, several pro-inflammatory mediators such as tumor necrosis factor-alpha (TNF-α) and IL-1β, secreted by alveolar macrophages, initiate the acute inflammatory cascade that triggers cell death and damage. Aside from PAMP/DAMP production, the recruitment of immune cells and activation of the nucleotide-binding domain leucine-rich repeat-containing receptor, pyrin domain-containing 3 (NLRP3), establish a pro-inflammatory positive feedback cascade (32, 34, 35) (Figure 1B steps 3 and 4). This localized inflammatory cell death could lead to a hyper-inflammatory microenvironment and spread to the vasculature, inducing leakage, edema, and pneumonia in COVID-19 patients (35, 37). Serum of COVID-19 patients is characterized by increased levels of the following: IL-2, IL-7, IL-10, TNF-α, protein monocyte chemoattractant-1 (MCP1; also known as C-C motif chemokine ligand 2 CCL2), granulocyte colony-stimulating factor (G-CSF), macrophage inflammatory protein 1 alpha (MIP1α; also known as CCL3), C-X-C motif chemokine ligand 10 (CXCL10), C-reactive protein (CRP), D-dimers and ferritin (19, 38–40).
2) The SARS-CoV-2 infection can also lead to the generation of adaptive immunity and neutralizing antibody (NAb). The virus-NAb complex can trigger Fc receptor (FcR)-mediated inflammatory response and acute lung injury. SARS-CoV-2 can infect cells that have FcRs, which provide the ability for antibody-mediated internalization. This mechanism can occur in macrophages, monocytes, or B cells even without ACE2 and TMPRSS2 expression, and especially during infection (41). The internalization of the virus–antibody immune complexes can also promote tissue damage and inflammation by activating myeloid cells *via* FcRs (42). Both primary and secondary responses culminate in the postulated pathogenesis of SARS-CoV-2 infection (32). Interestingly, it has been suggested that fatal COVID-19 is characterized as a cytokine release syndrome (CRS) induced by a cytokine storm and associated with adverse outcomes of acute respiratory distress syndrome (ARDS) (Figure 1B step 5) and high mortality rate (43, 44).

For mechanistic insights into the life cycle of SARS-CoV-2, the mouse hepatitis virus (MHV) represents a suitable comparator. MHV is a βCoV very similar to SARS-CoV, MERS-CoV, and SARS-CoV-2 (45). Therefore, an MHV animal model could contribute to the elucidation of the neuropathological mechanisms of SARS-CoV-2 in the following aspects: (1) MHV can invade and replicate in the CNS, triggering lesions in the white matter (45); (2) Infection with MHV induces meningoencephalitis in an acute stage and subsequently subacute chronic inflammatory demyelination in the brain and spinal cord (46); (3) CD4 and CD8 T lymphocytes, especially γδ T cells, play an important role in the MHV-induced demyelination process (47); (4) MHV can be translocated from the initial inoculation brain area to the spinal cord through the transit of viral particles in glial and neural cells, as well as by mechanisms that involve the fusion of lipid membranes (48); (5) After intranasal MHV-CoV inoculation in mice, the virus can access the CNS through the olfactory nerve and spread from this area to neuroanatomically interconnected structures such as the limbic system and the brainstem (49).

### Potential Neuroinvasive Pathways of SARS-CoV-2

There are four possible routes by which SARS-CoV-2 could enter the CNS: (1) the hematopoietic pathway and subsequent rupture of the blood-brain barrier (BBB); (2) *via* blood-cerebrospinal fluid (B-CSF); (3) transsynaptic viral spreading; (4) through the entry to circumventricular organs (CVO). In this section, we will discuss the four routes in greater detail.

**Figure 2:**
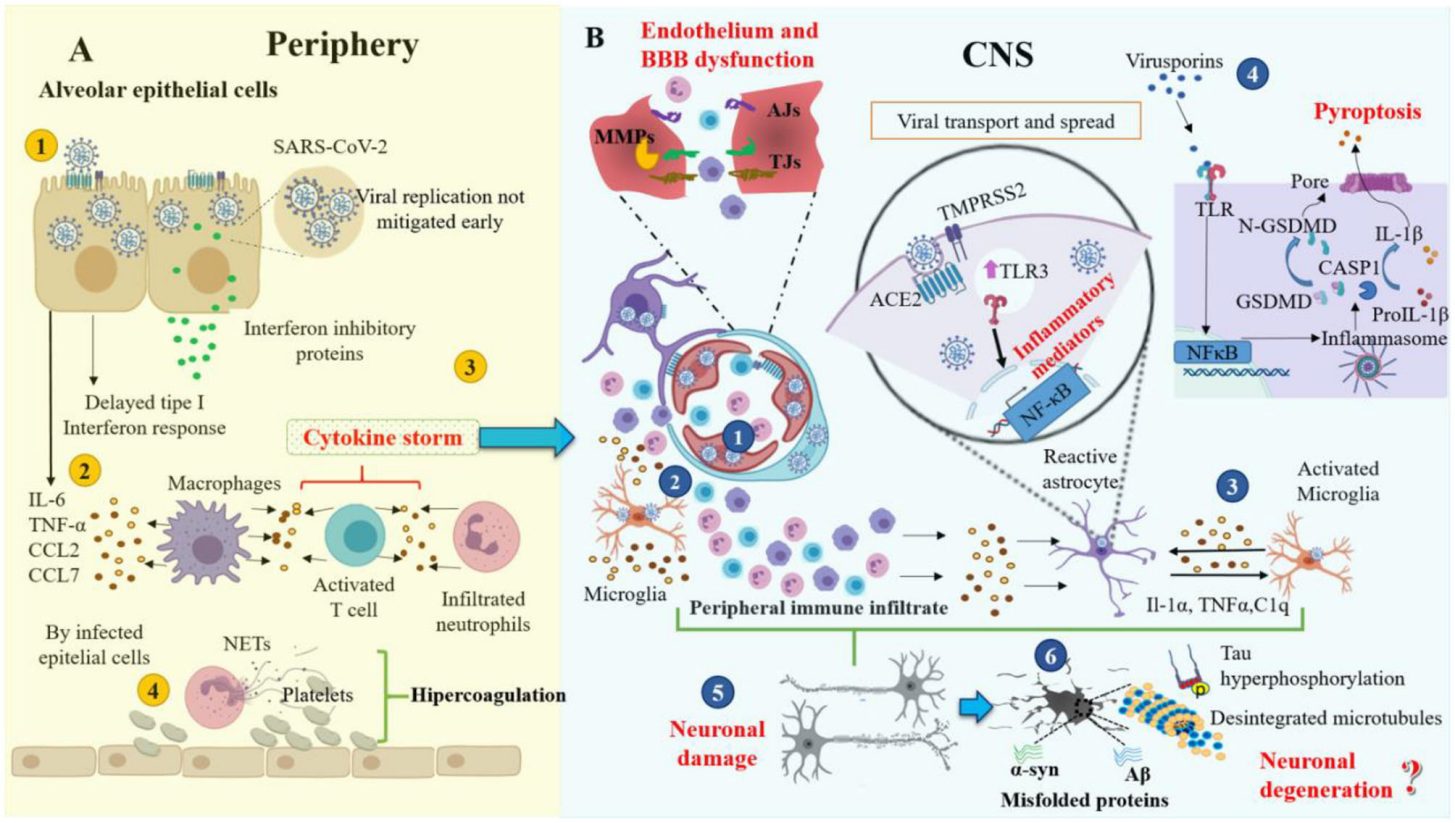
Schematic representation of the pathophysiological mechanisms of SARS-CoV-2. **(A)** Peripheral pathological events triggered by SARS-CoV-2 infection. **(B)** Possible CNS pathological mechanisms caused by the severe peripheral hyperinflammation associated with COVID-19. ACE2, angiotensin-converting enzyme 2 receptor; AJs, adherent junctions; Aβ; amyloid-beta; BBB, blood-brain barrier; C1q, the complement component 1q; CASP1, caspase1; CCL, chemokine (C-C motif) ligand; CNS, central nervous system; CXCL10, C–X–C motif chemokine 10; GSDMD, gasdermin-D; IL, interleukin; MMPs, metalloproteinases; NETs, neutrophil extracellular traps; NF-κB, Nuclear factor Kappa B; N-GSDMD, N-terminal gasdermin; NLRP3, nucleotide-binding domain-, leucine-rich repeat-containing receptor, pyrin domain-containing 3; TJs, tight junctions; TLR3, toll-like receptor 3; TNF-α, tumor necrosis factor-alpha; α-syn, alpha-synuclein.

**Figure 3:**
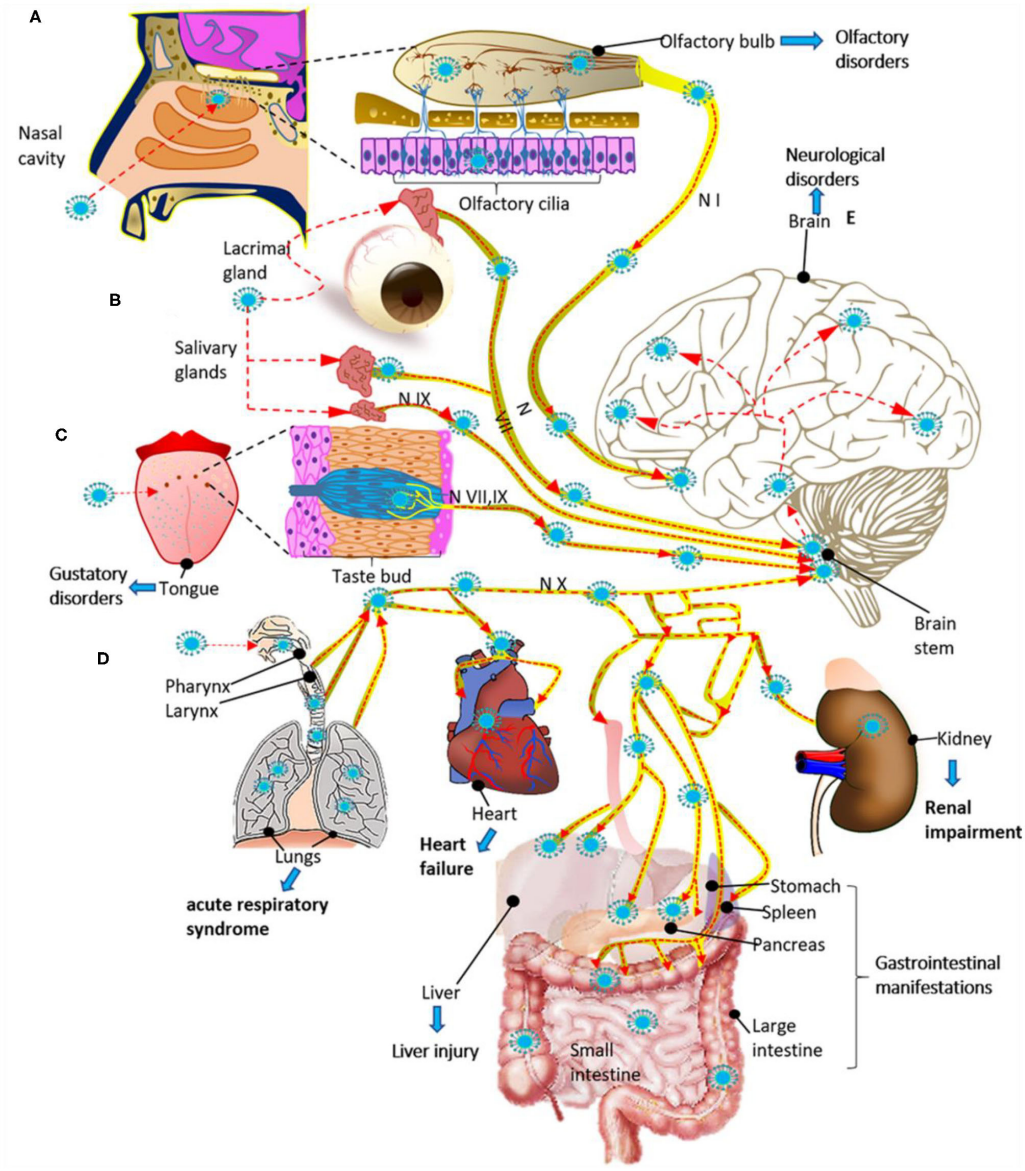
Potential routes for infection and spread of SARS-CoV-2 to systemic organs and the central nervous system through the cranial nerves (N). **(A)** SARS-CoV-2 could enter through the olfactory mucosa (causing anosmia), spread through the olfactory nerve (N I) and end in the olfactory cortex. **(B)** SARS-CoV-2 could also enter through the lacrimal and salivary glands, spread through the facial (N VII) and glossopharyngeal (N IX) nerves, and end in their respective brain stem nuclei. **(C)** The infection could spread from the taste buds (triggering ageusia) through the N VII and N IX nerves ending in the NTS located in the brain stem. **(D)** SARS-CoV-2 could also enter through the respiratory tract, reach the respiratory system and *via* the vagus nerve (N X), spread to other systemic organs innervated by this nerve, and end in the brain stem. **(E)** Finally, once the virus reaches the brain stem, it can spread to the brain through neuroanatomically interconnected pathways. The SARS-Cov2 infection can cause multiple organ dysfunction syndrome **(A–E)**. The red dashed arrows indicate the possible dissemination route for SARS-CoV-2 through the cranial nerves.

1) Coronaviruses access the bloodstream *via* the airway and infect immune cells, which may cross BBB facilitated by pro-inflammatory cytokines and chemokines (Figure 2B step 1). The mechanism by which infected immune cells cross the BBB may occur *via* intercellular adhesion molecule 1 (ICAM-1) mediated transport that is upregulated by TNF-α, followed by activation of matrix metalloproteinases (MMPs) such as MMP9, which specifically influences cellular leakage and membrane degradation (50). Besides, SARS-CoV-2 tropism, toward the CNS endothelial cells (BECs) favors BBB disruption; by entering the cytosol of the astrocyte *via* the receptor; the virus increases the release of pro-inflammatory cytokines, such as IL-2, IL-6, IL-7, IL-8, TNFα, CCL2, CCL3, CCL7, and CXCL10. The reactive astrocyte could lead to activation of microglia and the peripheral immune infiltrate such as macrophages, neutrophils, and lymphocytes (Figure 2B step 2) (51–54), which could end in neurotoxicity. DM, hypertension, and metabolic syndrome are risk factors for both contracting COVID-19 and a poor prognosis in patients. These comorbidities also contribute to vascular and BBB alteration (55), increase neuroinflammation, and exacerbate neuropathology (56).
2) The CSF circulation comprises both a directional CSF flow and a pulsatile to and from movement throughout the entire brain and which involves a local fluid exchange between blood, interstitial fluid, and CSF (57). It has been suggested that viral infection may occur *via* B-CSF and alter gene expression in the choroid plexus. This process activates the nuclear factor kappa (NF-kB), upregulates MMP9, and affects B-CSF permeability and immune cell trafficking (MMP8, TNFα, IL6, IL1B, MCP1, intercellular adhesion molecule 1 (ICAM1) (58), leading to a neuroinflammatory environment.
3) Another entry route to the CNS for SARS-CoV-2 could be through axonal transport and transneuronal spread from olfactory, gustatory, trigeminal, and vagal nerves, allowing the virus to infect the brainstem in the early stages of infection (Figures 3A–D) (52, 59). The transneuronal pathway is one of the potential routes that would allow SARS-CoV-2 to enter through the primary sensory neurons, which communicate with the mitral cells. Mitral cells have projections toward the ventricle and the medulla, and this favors the transfer of the virus from the cerebrospinal fluid toward the lymphatic system within the CNS and toward the PNS (60). The virus could also enter the CNS following the transneuronal olfactory bulb pathway and is reflected by changes at the level of the olfactory nerve, bulb, and cortex (61–63). It has been proposed that SARS-CoV-2 could spread retrogradely through transsynaptic transfer, using an exocytosis/endocytosis mechanism or *via* rapid axonal transport, which would move the virus along the microtubules to the neuronal soma (64). Supporting this hypothesis, it has been shown that some CoVs and other viruses such as rabies and hemagglutinating encephalomyelitis can enter and spread to the CNS *via* retrograde transsynaptic pathways (65–67), from peripheral nerve endings through membranous-coating-mediated endocytosis and exocytosis (66). Mechanisms have also been described by which viruses can enter and leave axons, both retrograde and anterograde, through coupled transport mediated by vesicles or separate transport which is not mediated by vesicles (68). For this reason, it would be interesting in the future to know if SARS-CoV2 uses transsynaptic transport and to trace neural circuits, using specific labeling techniques.
4) Finally, we suggest that SARS-CoV-2 might enter the CNS through CVOs. CVOs include the subfornical organ, the paraventricular nucleus, the nucleus tractus solitarius (NTS), and the rostral ventrolateral medulla, all of which express ACE2. Besides, these CVOs are highly vascularized and lack a BBB (69). Therefore, these areas would be more susceptible to the virus, triggering neurovascular damage, as we have discussed previously.

Once SARS-CoV-2 enters the CNS, it could bind to CNS cells, such as neurons, astrocytes, oligodendrocytes, and microglia (70), due to the presence of ACE2 (28, 71) and TMPRSS2 (72) receptors and probably *via* binding to other receptors (Table 1). It is important to highlight that the expression of ACE2 is low in the human brain, with a higher expression in certain areas such as thalamus and choroid plexus. ACE2 also has access to peptides in the circulation in the cerebrospinal and interstitial fluid, and it is present in pericytes and smooth muscle cells of human brain vessels (95). ACE2 receptors have been reported in other organs, mainly enterocytes, renal tubules, gallbladder, cardiomyocytes, male reproductive cells, placental trophoblasts, ductal cells, eye, and vasculature. In the respiratory system, its expression is limited (96). ACE2 plays a role in attenuating microvascular pathology and protecting against atherogenesis, endothelial dysfunction, thrombus formation, oxidative stress, and inflammatory cascades responsible for monocyte-endothelial cell interaction (71, 97).

**Table 1:** Receptors or proteins related to SARS-CoV-2 infection in the nervous system.

**Receptor or protein**	**Expression in nerve cells**	Neuroanatomic areas of gene expression^*^	**Pathological effects on the nervous system**	**References**
ACE2	Neurons, astrocytes, microglia, BECs, OLGs	PG, Acb, Hy, SC, Cd, SN, Cb, HiF, FroCx, Amg, Pu and ACC	The direct binding of SARS-CoV-2 to the ACE2 receptor could trigger microvascular dysfunction, disrupt coagulation processes, cause neuronal depolarization, and increase expression of glutamate and MMPs, resulting in neuroinflammation, seizures, and hemorrhages.	(56, 58, 69, 73–75)
TMPRSS2		PG, Hy, Cb, Amg, Cd, HiF, SN, Acb, ACC, FroCx, Pu and SC.	It acts as a co-receptor for ACE2 and cleaves S protein, facilitating viral binding to the ACE2 receptor and its activation. Therefore, it promotes the same effects described for ACE-2.	(76–78)
DPP4	Astrocytes (in murine)	FroCx, SC, ACC, PG, SN, Hy, HiF, Amg, Cb, Acb, Cd and Pu.	It is strongly associated with MERS-CoV. The murine models for DPP4 receptor infected with MERS-CoV have shown neuronal damage and peripheral immune infiltrates.	(79–81)
TLR4	Astrocytes, microglia	Cd, SN, Acb, Amg, Pu, SC, ACC, FroCx, y, HiF and Cb.	Molecular docking studies have demonstrated the binding of the native S protein of SARS-CoV-2 to TLR1, TLR4, and TLR6. However, TLR4 is most likely to recognize molecular patterns from SARS-CoV-2 to induce inflammatory responses. In CNS, it could promote the neuroinflammation environment.	(82, 83)
ATR1	Neurons, astrocytes	PG, SN, Hy, Cb SC,HiF, Cd, Acb, Pu, Amg, FroCx and ACC	It has been suggested that SARS-CoV-2 causes lung damage by increasing Ang II production. The hyperactivation of Ang II/ATR1/ACE signaling results in increased expression of pro-inflammatory cytokines, macrophage activation, and possibly BBB dysfunction.	(84–87)
ITGB1	Microglia	SC, PG, SN, Hy, HiF,Pu, Cd, Amg, FroCx, Acb, ACC and Cb.	It has been suggested that ITGB1 could bind to S protein through the RGD or KGE motif. ITGB1 mainly activates the MI3K/MAPK pathways, inducing an inflammatory response.	(24, 88, 89)
CatB and CatL	Microglia, neurons, astrocytes.	Cat B: FroCx, PG, SC, Cb, Hy, Acb, ACC, Cd, SN, Pu, HiF and Amg Cat: PG, SC, FroCx, Cb, SN, Hy, Cd, Acb, Pu, ACC, HiF and Amg	It has been suggested that S protein priming is partly dependent on the endosomal proteases, CatB and CatL. Nevertheless, TMPRSS2 is essential for viral entry into primary target cells and viral spread in the infected host. Also, CatB and CatL can contribute to the neuroinflammatory process.	(90, 91)
NLRP3	Microglia, astrocytes, neurons	SC, FroCx, Acb, Hy, SN, ACC, HiF, Amg, Cd, PG, Pu, Cb	To date, it is unclear if SARS-CoV-2 activates the NLRP3 inflammasome. However, SARS-CoV expresses at least three proteins (viroporins) that activate the NLRP3 inflammasome: envelope (E), ORF3a, and ORF8b. The NLRP3 inflammasome activation could trigger inflammatory cell death.	(92–94)

*Neuroanatomic areas and nerve cells in which these receptors or proteins are expressed and their possible neuropathological effects*.

*Acb, nucleus accumbens; ACC, anterior cingulate cortex; ACE2, angiotensin-converting enzyme 2; Amg, amygdala; ATR1, angiotensin receptor type 1; BBB, blood-brain barrier; BECs, brain endothelial cells; Cat, cathepsin; Cb, cerebellum; Cd, caudate nucleus; DPP4, dipeptidyl peptidase-4; FroCx, frontal cortex; HiF, hippocampal formation; Hy, hypothalamus; ITGB1, integrin subunit beta 1; KGE, Lys-Gly-Glu; MAPK, Mitogen-Activated Protein Kinases; MERS-CoV, Middle East Respiratory Syndrome Coronavirus; MI3K, myo-inositol 3-kinase; MMPs, matrix metalloproteinases; NF-kB, nuclear factor kappa B; NLRP3, nucleotide-binding domain-, leucine-rich repeat-containing receptor, pyrin domain-containing 3; OLGs, oligodendrocytes; PG, pituitary gland; Pu, putamen; RGD, Arg-Gly-Asp; SARS-CoV-2, severe acute respiratory syndrome coronavirus 2; SC, spinal cord (cervical c-1), SN, substantia nigra; TLR, Toll Like Receptor; TMPRSS2, transmembrane protease serine 2*.

* *The GTEx Analysis Release V8 (dbGaP Accession phs000424.v8.p2) was used to obtain the gene expression data (from highest to lowest expression) in several brain areas*.

SARS-CoV-2 interaction with ACE2 could cause astrogliosis and microgliosis, increase BBB permeability, allowing monocyte and leukocyte infiltration to the CNS in multiple brain regions (98, 99). These areas include the olfactory bulb, choroid plexus, cerebral cortex, caudate/putamen, ventral striatum, thalamus, hypothalamus (paraventricular nuclei), spinal cord, hippocampus, frontal cortex (52, 95, 100), substantia nigra, middle temporal gyrus (64, 101), and other brain areas (Table 1). Since many viruses have neurotropic properties (102), SARS-CoV-2 could spread through neuroanatomically interconnected pathways (103) and lead to nerve cell dysfunction and neurodegeneration in the CNS.

### Can the Systemic Inflammation by COVID-19 Trigger Neurovascular Disturbance?

In this section, we highlight evidence at the systemic level and locally in the respiratory tract tissue of patients with COVID-19. Since this virus induces lung pathology, the detailed information of other organs and systems such as the CNS has yet to be fully investigated. Therefore, the nerve signaling pathways proposed here are based on the systemic evidence from similar viruses.

Channappanavar and Perlman focused on the systemic immune response against pathogenic human coronaviruses such as SARS-CoV and Middle East respiratory syndrome CoV (MERS-CoV). They proposed that a dysregulated immune response in the host is responsible for triggering the pulmonary pathology and fatal clinical manifestations (104).

On the other hand, high levels of viral replication in the host could contribute to tissue damage. Two mechanisms might be responsible: first, the delayed induction of interferon responses and second, the production of interferon inhibitory proteins by the human CoVs. Therefore, early unmitigated viral replication could be responsible for the high and exaggerated production of cytokines and chemokines by infected alveolar epithelial cells, macrophages, and leukocytes infiltrated into the lung tissue, which leads to severe damage (Figure 2A steps 1–3) (104). Other investigators have concluded that COVID-19 is characterized by an extreme hyper-inflammatory process followed by hyper-coagulation (Figure 2A step 4) (105).

CRS is a systemic inflammatory response that can be triggered by SARS CoV-2 infection, characterized by a drastic increase in the levels of the pro-inflammatory cytokines (Figure 2A) (106). The CRS may induce a MODS in COVID-19 patients, which is characterized by acute failure of different organs such as the liver, kidney, heart, and as well as hematological, gastrointestinal, and neurological disorders (Figure 3) (107). Besides, it has been proposed that patients who died from severe COVID-19 have a significant endothelial affectation or “endothelitis,” which may be associated with MODS. It has been suggested that endothelial dysfunction in several organs may be triggered by the interaction between SARS-CoV-2 with ACE2 receptors that express endothelial cells and the subsequent inflammatory response (108, 109). This inflammatory response can contribute to increased vascular permeability, edema, and the synthesis of coagulation factors (110). From a meta-analysis, it has been reported that levels of D-dimer, an indicator of fibrinolysis, have been reported following severe infection by COVID-19 (111). The formation of clots could result in the occlusion of blood vessels and cerebral arteries, which can lead to cerebral venous thrombosis. Therefore, we assume that some of the symptoms and even neurological complications may be caused by the systemic cytokine storm and subsequent endothelium and BBB dysfunction (Figure 2). In this way, systemic hyper-inflammation caused by maladaptive innate immunity may trigger neurovascular function damage, a BBB rupture, and activate the CNS innate immune signaling pathways (112). This BBB disruption could promote immune cell infiltration (113) (Figure 2B steps 1 and 2). The intracerebral cytokine storm also could contribute to the BBB rupture (114, 115), leading to a vicious cycle of increasing pathology. These events may also be responsible for developing other neuropathies such as necrotizing encephalopathy or Guillain-Barré syndrome (GBS) (116, 117). The coagulopathy observed in COVID-19 could make patients prone to thrombotic cerebrovascular or bleeding events (118).

On the other hand, microglia and astrocytes are the main cell lineages that mediate immunological processes within the CNS. Thus, microglia, the macrophage of the CNS par excellence, can also promote states of hyper-inflammation that exacerbate hypercoagulation by infiltration of professional immune cells and coagulation elements. Nevertheless, we hypothesize that SARS-CoV-2 could activate the microglia and, subsequently, induce the reactivation of A1 astrocytes *via* secreting IL-1α, TNF, and the complement component 1q (C1q), as occurs in other neurological diseases (119) (Figure 2B step 3). Besides, exposure to the viruses or their components promotes the expression and activation of Toll-like receptors (TLR) in astrocytes. This signaling promotes the production and release of pro-inflammatory mediators and induces inflammatory responses in the CNS (Figure 2B step 3), eliminating the pathogen as demonstrated for Flavivirus infections (82, 120). Therefore, this pathological signaling causes neuronal degeneration and dysfunction of the nerve cells short or long term.

Viroporins belong to a family of small transmembrane proteins that include CoV protein E (121, 122), which could generate neurotropism of SARS-CoV. According to previous studies, viroporins can promote the activation of the NLRP3 inflammasome (123, 124). The NLRP3 inflammasome is a subcellular multiprotein complex that is highly expressed in several nerve cells and CNS areas (Table 1). Activation of the NLRP3 occurs after infection by the influenza A virus and SARS-CoV (125) (Figure 2B step 4). After NLRP3 inflammasome activation, caspase-1 and other non-canonical inflammasome caspases (caspase-4, caspase-5, or caspase-11) activate gasdermin-D (GSDMD), which subsequently forms pores in the cell membrane. These pores facilitate the secretion of IL-1β and IL-18 and, importantly, they also enable the simultaneous influx of Na^+^ and water molecules, facilitating neuroinvasion by causing excessive cellular swelling, membrane rupture, and subsequent pyroptosis, an inflammatory form of cell death (126, 127). Therefore, it is possible that pyroptosis may occur in nerve cells (Figure 2B step 4).

### Neurological and Neuropsychiatric Manifestations, Diagnostic, and Treatment in Patients With COVID-19

The respiratory symptoms caused by the SARS-CoV-2 virus are still the most readily identified and studied. However, neurological manifestations are beginning to take on unquestionable importance, mainly in the critically affected patient. Our understanding of the long-term neurological symptoms is limited and presents a real challenge (13, 14, 62, 128). A physiopathological explanation for the neurological and neuropsychiatric manifestations of COVID-19 has yet to be found. Although there are hypotheses about the direct effects of SARS-CoV-2 on the CNS and PNS, evidence suggests that these effects can be attributed to other causes such as: (1) the impact of the systemic inflammatory response caused by the virus and (2) the underlying comorbidities of the patients (62, 129, 130). Patients with mild COVID-19 have been reported to have non-specific neurological disorders such as headache and myalgias, dizziness, dysgeusia, and anosmia with variations in their prevalence (Table 2) (13, 14).

**Table 2:** SARS-CoV-2 infection in the central and peripheral nervous system: clinical manifestations, mechanism of pathogenicity, laboratory, and clinical findings and suggested treatment.

**Clinical manifestation**	**The probable mechanism of pathogenicity**	**Laboratory and/or clinical alterations**	**Treatment or recommendations**	**References**
**Central nervous system**				
**Headache**: occurs in ~70% of patients, with an average duration of 3 days.	(1) Direct viral invasion of the trigeminal nerve endings in the nasal or oral cavity. (2) An increase in the levels of peptides related to the circulating calcium gene has been linked to the trigeminal vascular activation.	The use of neurological and laboratory imaging techniques is only recommended if the headache is associated with focal neurological symptoms.	(1) NSAIDs and steroids are not recommended as they can exacerbate COVID-19 symptoms. (2) Anticonvulsants may offer benefits.	(131–133)
**Delayed awakening**: has been observed in some patients after ventilation for COVID-19-related ARDS.	A relationship between posterior circulation inflammation and brainstem function may be related to altered consciousness.	(1) Brain MRA: an increase in the abnormal contrast has been observed in the arterial wall associated with endotelialitis. (2) EEG: non-specific changes have been observed. (3) In serum and CSF: oligoclonal bands have been observed.	The use of IV methylprednisolone has been proposed for 5 days, followed by decreasing doses of prednisone.	(130)
**Encephalopathy**: One study Liotta et al. (134) determined that it was present in about a third of patients, and was associated with increased mortality.	It has been proposed that they may be involved in toxic-metabolic processes such as hypoxemia, ROS production, and organ failure.	MRI: intensity changes in the leptomeningeal spaces, in the mesial temporal lobe, and the hippocampus, as well as frontotemporal hypoperfusion.	The use of low potency antipsychotic agents and alpha-2 agonists has been proposed to control psychomotor agitation.	(134–136)
**Ischemic stroke even**t: is a life-threatening complication and is associated with cardioembolic events.	(1) Elevated inflammation, DIC, and hypoxia have been associated with a state of hypercoagulability. (2) Complement activation is associated with microvascular damage leading to thrombotic injury.	(1) The neuroimaging patterns observed are extensive vessel thrombosis, embolism, or stenosis, followed by affected multiple vascular territories. (2) Laboratory studies have revealed an increase of D-dimer, fibrinogen, antiphospholipid antibody levels.	Prophylactic or therapeutic anticoagulation therapy, as well as thrombectomy, have been recommended.	(62, 137)
**Hemorrhagic stroke:** has been attributed to COVID-19 and risk factors as anticoagulation, trauma, and hypertension.	Lupus anticoagulant and antiphospholipid antibodies have been suggested to play a role in its pathophysiology.	Imaging studies have revealed microhemorrhage foci, hematomas larger than 5cm, surrounding edema, and even descending hernia.	Reduce risk factors that affect hypertension, aneurysm, and states of anticoagulation.	(62)
**Peripheral nervous system**				
**Olfactory disorders**: present around 86% of COVID-19 patients: anosmia (79%), hyposmia (20.4%), phantosmia (12%), and parosmia (32%) Lechien et al. (138).	(1) Nasal epithelial damage is characterized by a reduced number of ORs and abnormal dendrites that do not reach the epithelial surface or lack sensory cilia. (2) Substitution of ONE with metaplastic squamous epithelium. (3) Inflammation can lead to impairment of ORs and also damage of olfactory neurons.	MRI has shown abnormalities in the signaling of one or both olfactory bulbs, edema of the olfactory bulb, and microhemorrhage in one of the olfactory bulbs.	The most widely used treatments for olfactory dysfunction are saline nasal irrigations, nasal corticosteroids, oral corticosteroids, vitamins, and trace elements.	(138–141)
**Gustatory disorders**: present around 88% of COVID-19 patients: hypogeusia (79%) or dysgeusia (21%) Lechien et al. (138)	(1) Diffuse expression of ACE2 receptors (modulation of taste perception) in the oral mucosa, particularly in the tongue. (2) SARS-CoV-2 can bind to sialic acid receptors, accelerating the degradation of taste particles.	Recent evidence suggests that imaging or laboratory studies are not usually done on patients who only manifest gustatory disorders.	Treatment for these disorders has not been established; however, l-carnitine or trace elements and vitamins have been used.	(138, 142)
**Neuromuscular disorders:** myalgia and fatigue affect between 44 and 70% of patients. About 10% of patients have a skeletal muscle injury.	(1) SARS-Cov-2 could trigger viral myositis. (2) Alteration in the expression of ECA2 in skeletal muscle. (3) Skeletal muscle damage from cytokine storm.	(1) Elevated serum creatine kinase levels. (2) Muscle injury has been associated with multiple organ damage, such as liver dysfunction (increased levels of LDH, ALT, and AST) and kidney (increased levels of blood urea nitrogen and creatinine).	The use of corticosteroids has resulted in benefits.	(129, 143)
**Guillain-Barre Syndrome GBS:** has been associated with COVID-19. Interestingly, the interval between the onset of COVID-19 symptoms and the first symptoms of GBS has ranged from 5 to 10 days.	(1) It has been proposed that it serves the same mechanisms as typical GBS, consisting of demyelination of peripheral nerve roots. (2) Peripheral nerve damage can be caused by the immune response to SARS-CoV-2, driven by the production of autoreactive antibodies (anti-ganglioside).	(1) Hematological and biochemical examinations have shown leukocytosis, leukopenia, thrombocytosis, thrombocytopenia, and elevated levels of CRP. (2) CSF tests have shown cytological dissociation of albumin. (3) EMG has been associated with a demyelinating process. (4) MRI has revealed an enhancement in the caudal nerve roots and the facial nerve.	The therapeutic protocol to GBS associated with COVID-19 has been typically used for this pathology: IV immunoglobulin or plasma exchange, supportive care, and antiviral drugs.	(144–146)

*ACE2, angiotensin-converting enzyme 2; ALT, alanine transaminase; ARDS, acute respiratory distress syndrome; AST, aspartate aminotransferase; CNS, central nervous system; CRP, C-reactive protein; CSF, cerebrospinal fluid; DIC, disseminated intravascular coagulation; EEG, electroencephalography; EMG, electromyography; GBS, Guillain-Barre syndrome; IV, intravenous; LDH, lactate dehydrogenase; MRA, magnetic resonance angiography; MRI, magnetic resonance imaging; NSAIDs, non-steroidal anti-inflammatory drugs; ONE, olfactory neuroepithelium; ORs, olfactory receptors; ROS, reactive oxygen species*.

Some studies point to headache as the most common neurological symptom and often as the only symptom of COVID-19 (129, 147). However, other authors have defined the headache as a consequence of systemic disease. It has been suggested that the chronic release or exposure of vasoactive peptides such as Calcitonin Gene-Related Peptide (CGRP; pain and migraine-related peptide) (148) can activate trigeminal sensory fibers and thus modulate the transmission of impulses related to headache (149). Besides, in COVID-19, a close link between cytokine storm and headache has been proposed, due to the release of the vasoactive peptides (150).

In contrast, for hospitalized patients, encephalopathy with neuropsychiatric manifestations such as delirium and agitation have been observed. There has also been a considerable increase in the reports of patients with neuromuscular diseases, among which are GBS with some of its variants and several rhabdomyolysis cases. However, it has not been possible to find a clear relationship between these manifestations and COVID-19. An increasing incidence of neurological manifestations has been observed in COVID-19-infected patients that have been associated with severe health conditions and prolonged hospital stays (129, 134). However, there are also reports of neurological manifestations in outpatients with COVID-19 infection (129, 138). Therefore, it is difficult to determine the incidence of each of the neurological manifestations due to the different screening methods applied for each reported case.

Neurological disorders such as multiple sclerosis (MS), encephalopathy, and GBS, have been associated with SARS-CoV2. In some cases, CNS demyelination has occurred shortly after SARS-CoV-2 infection, suggesting a causal relationship between these two pathologies (151). Viruses, such as the Epstein-Barr virus (EBV), have been linked to MS, with high titers of EBV antibodies found in MS patients. Viral induced demyelination could be a direct result of viral infection of oligodendrocytes, which leads to cell death and myelin degeneration, or to the exacerbated inflammatory response caused by virus replication (152, 153). The cytokine storm caused by SARS-CoV-2 may cause the activation of glial cells and the start of the demyelination process (154). Conversely, other studies suggest that SARS-CoV-2 could act as an accelerating factor for MS but not the trigger for the disaese (155). Likewise, several case reports have reported the appearance of GBS after SARS-CoV-2 infection (156–158). Although hypoxic/metabolic changes caused by intense inflammatory response against the virus together with the presence of comorbidities, may result in encephalopathy (159) there is still insufficient evidence to prove that SARS-CoV-2 virus infection invades the CNS directly to provoke encephalopathy (151). In the same way, despite the complications associated with SARS-CoV-2 infection in patients with GBS, there is no clear evidence yet that COVID-19 initiates GBS (151).

Cerebral events have been associated with SARS-CoV-2 patients, with cerebral ischemic events being the most frequent. Cerebral hemorrhages and microhemorrhages are also noticed (62, 144) (Table 2).

As discussed previously, the cranial nerves might also be susceptible to a direct or indirect injury caused by SARS-CoV-2. According to a recent study, about 86 and 88% of patients with COVID-19 develop olfactory and gustatory alterations, respectively (138). These findings might be specific for SARS-CoV-2 infection and be useful to distinguish them from other causes. Interestingly, the presence of these dysfunctions can precede the onset of respiratory symptoms (160, 161) and may predict a mild clinical course of the disease (162) (Table 2). Besides, it has been proposed that pericytes of the olfactory bulb, which express high levels of the ACE2 receptor, may be responsible for triggering the cytokine storm and thus causing olfactory disorders in COVID-19 patients (163).

On the other hand, neuroimaging data could help us understand the pathological effects of SARS-CoV-2 in the CNS and PNS. Unfortunately, published brain imaging findings from confirmed COVID-19 patients are currently scarce and limited to small case series. However, it has been possible to correlate them with the potential pathophysiological mechanisms involved. For example, in a recent study, it was shown that patients with COVID-19 presented multifocal petechial hemorrhages associated with BBB rupture (164). In another study, microhemorrhages and macrohemorrhages were associated with posterior reversible encephalopathy syndrome (165) (Table 2). Although the underlying mechanism of brain abnormalities detected through neuroimaging remains to be understood, these findings provide further evidence that CNS damage can occur in COVID-19 patients. The correct understanding of pathophysiological mechanisms of neurological manifestations may reveal potential therapeutic targets (Table 3). Depending on the neurological complications associated with COVID-19, treatments would need to be adjusted accordingly (Table 2).

**Table 3:** Current drugs used against COVID-19.

**Drug**	**Mechanism of action**	**Results of clinical case reports**	**Adverse effects**	**Dose**	**References**
**Antiviral drugs**					
Remdesivir	The prodrug, belonging to the group of nucleotide analogs, generates an active metabolite capable of entering cells and inhibits viral RNA polymerase. Inhibitory capacity against SARS-CoV-2 *in vitro* has been observed^*^.	Decreased recovery time, disease progression, as well as mortality compared to placebo.	-Infusion-related hypotension - Hepatotoxic - Nephrotoxic - Gastrointestinal symptoms	-First dose of 200 mg -100 mg/day for 5–9 days.	(166)
**TMPRSS2 antagonist**					
Camostat	Produces GBPA, that inhibits many of the serine proteases that SARS CoV and SARS-CoV-2 use for virus-to-host cell membrane fusion, like TMPRSS2^**^.	Reduces the likelihood of serious infection, as well as morbidity and mortality.	-Eruption - Pruritus - Oedema - Urticaria	It has been used at different doses in humans and other pathologies. e.g., : 200 mg every 8 h	(167)
Nafamostat mesylate	Inhibited SARS-CoV-2 S protein-mediated entry into host cells with about 15-fold-higher efficiency than camostat, with a 50% effective concentration.	In combination with Favipiravir has shown a decrease in mortality.	Hyperkalemia	0.2 mg per kg/hour by continuous IV infusion, for 14 days.	(168, 169)
**Monoclonal antibodies**					
Tocilizumab	IL-6 receptor antagonist	May reduce the hospital stay, the need for ICU admission, and the need for invasive mechanical ventilation.	- Increased risk of secondary infections. - Hypersensitivity reactions - Neutropenia and thrombocytopenia - Hepatotoxicity	>75 kg: 600 mg single dose <75 kg: 400 mg single dose^***^	(170)
Anakinra	IL-1 receptor antagonist	Reduced both needs for invasive mechanical ventilation and mortality in severe COVID-19 patients.	- Elevation of liver enzymes three times higher than their reference value. - Possible thromboembolic events.	100 mg every 6 h for a maximum of 15 days.	(171, 172)
Mavrilimumab	Binds to GM-CSFRα^****^ and disrupts downstream signaling.	Fast clinical improvement, decrease both the need for mechanical ventilation and mortality.	No adverse reactions to the infusion were observed.	6 mg/kg single dose.	(173)
**Steroids**					
Dexamethasone	- Anti-inflammatory action. - Inhibits phospholipase A2 and, consequently, prostaglandin, thromboxane, and leukotriene synthesis - Suppresses leukocyte migration - Recovers the BBB by upregulation of ZO-1 tight junction protein	Decreased mortality in patients requiring oxygen therapy and mechanical ventilatory support when treatment is initiated 7 days after symptom onset.	- Hyperglycemia - Increased risk of bacterial and fungal infections.	6 mg/day for 10 days.	(174)

*GBPA, 4-[4-guanidinobenzoyl-oxy] phenylacetic acid; GM-CSF, Granulocyte-macrophage colony-stimulating factor (GM-CSF); TMPRSS2, Transmembrane serine protease 2*.

* *Inhibitory activity against SARS-CoV-1 and MERS-CoV has been demonstrated*.

** *The high expression of TMPRSS2 in different brain areas could be a potential therapeutic target for neurological manifestations and complications*.

*** *According to safety criteria and clinical trial data*.

**** *GM-CSF is a cytokine with a cardinal role in inflammation modulation. Ligand binding to the GM-CSF receptor-α (GM-CSFRα) activates multiple pro-inflammatory pathways and, in macrophages and neutrophils, results in increased secretion of pro-inflammatory cytokines*.

### Neurohistopathological Findings by COVID-19

The histopathological analysis of nervous tissue of patients who presented neurological complications and died due to COVID-19 is undoubtedly precious to our understanding of the pathophysiology and potential therapeutic strategies (Table 4).

**Table 4:** Neurohistopathological findings in patients infected with SARS-CoV-2 and their association with neurological manifestations.

**Characteristics of the patients**	**Tissue and PMI**	**Histopathological findings**	**Neurological manifestations**	**References**
**Central nervous system**				
*n =* 18 age range: 53–75 years comorbidities: AF, ALL, BPH, CAD, CKD, COPD, DM, ESRD on HD, EtOH use disorder, HF, HTN, ILD, MGUS, NHL, OCD, OSA, PPV, PVD, RA-SLE.	Inferior-frontal lobe with olfactory tract/bulb, corpus callosum, hippocampus, occipital lobe, anterior basal ganglia, thalamus, cerebellum, midbrain, pons, and medulla. PMI: NS.	Acute hypoxic-ischemic injury with neuronal loss in the cerebral cortex, hippocampus, and cerebellar Purkinje cell layer. Arteriolosclerosis with perivascular rarefaction, a microglial nodule, and perivascular inflammation with scattered microglia were also detected.	It is associated with the confusional state, myalgia, headache or, hypogeusia.	(175)
*n =* 6 age range: 58–82 years comorbidities: EtOH use disorder, HTN, COPD, CKD, PHT, PVD, CAD, AF.	Hippocampus, neocortex, cerebellum, and brainstem nuclei. PMI: NS.	Lymphocytic panencephalitis and meningitis. Neuronal cell loss and axon degeneration in the dorsal motor nuclei of the CN X and V, NTS, dorsal raphe nuclei, and medial longitudinal fasciculus.	Associated with altered consciousness.	(176)
*n =* 1 age: 73 years commorbidities: DM and HTN.	Cortex, hippocampus, amygdala, striatum. PMI: NS.	Cerebellar hemorrhage, acute infarcts, global hypoxic changes with scattered hypereosinophilic shrunken neurons in the cerebral cortex, striatum, thalamus, amygdala, hippocampus, and the Purkinje cell layer.	Headache, nausea, vomiting, and loss of consciousness.	(177)
**Cranial nerves and peripheric nervous system**				
*n =* 33 age range: 67–79 years commorbidities: DM, HTN, CVD, HLD, CKD, PS and dementia.	Olfactory mucosa, bulb and tuber, oral mucosa, trigeminal ganglion, medulla oblongata, and cerebellum. PMI: NS.	High levels of viral SARS-CoV-2 RNA (RT–qPCR) and protein within the olfactory mucosa. Lower levels were found in the cornea, conjunctiva, and oral mucosa; and in only a few COVID-19 autopsy cases, the cerebellum was positive for SARS-CoV-2.	Alterations of smell and taste perception, impaired consciousness, headache, and behavioral changes	(178)
*n =* 2 age: 51 and 94 years commorbidities: COPD, IHD and AML	Glossopharyngeal, vagal nerves and other brain areas. PMI: 3.3 days	SARS-CoV-2 viral proteins mapped to isolated cells.	Ageusia	(179)
*n =* 21 age range: 41–78 years commorbidities: DM, CVD, COPD, asthma, ASM and AHM.	Olfactory bulbs, NTS and other brain areas. PMI: NS.	Extensive inflammation and infiltrating immune cells.	Anosmia and dampening of the respiratory system.	(180)

*AF, atrial fibrillation; AHM, active hematological malignancy; ALL, acute lymphoblastic leukemia; AML, acute myeloid leukemia; ASM, active solid malignancy; BPH, benign prostatic hyperplasia; CAD, coronary artery disease; CKD, chronic kidney disease; CN, cranial nerves; COPD, chronic obstructive lung disease; CVD, cardiovascular disease; DM, diabetes mellitus; ESRD on HD, end stage renal disease on dialysis; EtOH use disorder, alcohol use disorder; HF, heart failure; HLD, hyperlipidemia; HTN, hypertension; IHD, ischaemic heart disease; ILD, interstitial lung disease; MGUS, monoclonal gammopathy of undetermined significance; n, number of patients; NHL, non-Hodgkin lymphoma; NS, not specified; NTS, nucleus tractus solitarius; OCD, obsessive compulsive disorder; OSA, obstructive sleep apnea; PHT, pulmonary hypertension; PMI, postmortem interval; PS, prior stroke; PVD, peripheral vascular disease; RA-SLE, rheumatoid arthritis - systemic lupus erythematosus; RT-qPCR, reverse transcription-quantitative polymerase chain reaction*.

Solomon et al. analyzed nervous tissue from 18 patients infected with SARS-CoV-2 who had also presented with certain comorbidities such as DM, hypertension, cardiovascular disease, hyperlipidemia, chronic kidney disease, and dementia. The histological examination (Table 4) revealed a greater number of copies of SARS-CoV-2, acute hypoxic-ischemic injury, neuronal loss, and perivascular inflammation in several brain areas, and even pathological features of Alzheimer's disease (AD) were observed (175). A different case report described the brain from a 73-year-old man with unspecified neurological manifestations (Table 4), hypertension and DM and positive for SARS-CoV-2 with cranial computed tomography. The results showed right cerebellar intra-parenchymal hemorrhage, edema, medulla compression, and tonsillar herniation. After 18 h without improvement, the patient died of palliative extubation. Brain histopathology revealed severe global hypoxic changes with scattered hypereosinophilic shrunken neurons in several brain areas and mild perivascular inflammatory infiltrates (Table 4) (177). This study also suggested a preference of the virus to the cerebellar Purkinje cell layer. In addition, astrogliosis was noted in the superior frontal and orbital cortices, while microglial activation in the cortex was not evident (177).

A study focused on describing the SARS-CoV-2 tropism within the olfactory mucosa to the CNS examined autopsy material from 33 patients positive for the virus. The authors showed viral RNA for SARS-CoV-2 within the olfactory mucosa sampled directly beneath the cribiform plate. They also found viral RNA in anatomically distinct regions such as cornea, conjunctiva and oral mucosa. Using immunohistochemistry, *in situ* hybridization, and electron microscopy, they suggested that SARS-CoV-2 neuroinvasion to the CNS occurs *via* axonal transport, thus explaining the well-documented neurological symptoms (Table 4) (178). The authors also proposed that SARS-CoV-2 infection in the cerebellar region may occur by the migration of the virus-carrying leukocytes across the BBB, without directly connecting this area to the olfactory mucosa.

Other cranial nerves have been considered as the route for entrance of the virus to the CNS (Figure 3) (181, 182). Regarding ageusia, the pathogenesis may involve an alteration in the glossopharyngeal, facial, vagus nerve, or the nucleus tractus solitarii (NTS), at the brainstem level (183). In an immunohistochemistry analysis of the cranial nerves from two individuals, SARS-CoV-2 and viral proteins were found within the medulla oblongata and in both glossopharyngeal and vagal nerves from the lower brainstem (179), suggesting these areas as a potential route for virus entry into the CNS and peripheral tissue (Table 4).

Other authors found hypereosinophilia or nuclear and cytoplasmic condensation of neurons in the cerebrum and cerebellum of severe COVID-19 patients due to hypoxic brain changes (Table 4). The olfactory bulb histopathological analysis showed many activated microglia with enlarged bodies and T-cell extravasation into the parenchyma and elevated levels of reactive astrocytes (180). Interestingly, the NTS showed astrogliosis and a massive microglial activation with the formation of a microglia nodule and T cells in the leptomeninges of the medulla oblongata (180). This implies extensive inflammation in this area, which results in a dysregulation of the respiratory system (184). One of the main limitations of these studies is that it is not clear whether the histopathological findings are the result of patients' comorbidities/aging, or due to SARS-CoV2 neuro-infection. Further investigations are necessary to make the corresponding comparison with healthy subjects with appropriate age ranges and to understand the neuropathological mechanism of SARS-CoV-2 and its relationship with neurological manifestations and patient comorbidities.

### The Potential Role of SARS-CoV-2 in the Pathogenesis of Neurodegenerative Diseases

The SARS-CoV-2 neurotropism has already been documented in several reports (Table 2) (185–187). However, it remains unknown whether SARS-CoV-2 contributes to neurodegenerative pathogenesis. It has been hypothesized that viruses can cause neurological problems by affecting neurotransmitter release, lysing the cells, inducing apoptosis, commanding neuronal transcriptional pathways or indirectly activating the immune response (188).

Neuroinvasive animal CoVs, such as the porcine hemagglutinating encephalitis virus (PHEV) or MHV have been shown to induce different types of neuropathology. Similarly, human CoVs, such as HCoV-229E and HCoV-OC43, have been implicated in establishing or exacerbating neurodegenerative diseases (189–192).

AD is the most common cause of dementia among the elderly. Neuropathological hallmarks of AD include the neurofibrillary tangles, consisting of intraneuronal and hyperphosphorylated tau, and the extracellular accumulation of amyloid β-peptide (Aβ) in the brain parenchyma in the form of neuritic plaques (193, 194). The neurovascular unit (NVU) and BBB dysregulation are also critical pathophysiological events in neurodegenerative diseases, including AD. Previous studies have suggested a relationship between AD and infectious agents. *Chlamydia pneumoniae, Helicobacter pylori, Borrelia burgdorferi*, and herpes simplex virus have been reported in post-mortem AD brain (195) and a relationship between virus infection and Aβ has been suggested. Soscia et al. noticed that Aβ exerted antimicrobial activity against relevant microorganisms and was modulated in response to some environmental stressors. Thus, transient viral infection could initiate or accelerate Aβ accumulation in the brain and neuronal damage (Figure 2B steps 5 and 6) (196). ACE2 can induce an increase of nitric oxide (NO) in the brain, which becomes neurotoxic. NO and other reactive species, which could be produced as a consequence of viral internalization and impairment of cell organelles (mitochondria, lysosomes), could, in turn, increase misfolding and aggregation of cellular proteins (197).

It has been proposed that SARS-CoV-2 could increase the hyperphosphorylation of tau in the axonal region (52, 198–200), promoting disassembly of microtubules and, subsequently, neuronal degeneration (Figure 2B steps 5 and 6) (201). Likewise, it has been suggested that persistent CoV infections can induce a neuroimmune response and a pro-inflammatory state, and activate glial cells (202). Microglial cells may be chronically activated by a single stimulus, such as pathogen infection, resulting in slow and progressive neuronal loss through multiple neurotoxic factors (203). Interferon (IFN), which has a role in mediating AD pathology, directly activates microglia and stimulates a pro-inflammatory response derived from SARS-CoV-2 infection (204). Therefore, it seems that the neurotropism of SARS-CoV-2 can lead to the activation of microglial cells, trigger chronic neuroinflammation, and finally, neurodegeneration (Figure 2B).

Finally, it has been found that ACE2 is upregulated in the cerebral vasculature of dementia cases. ACE2 increases the intracellular level of angiotensin 2, causing vasoconstriction and promoting brain degeneration (205). Buzhdygan et al. demonstrated that S1 could promote BBB alteration in an advanced 3D microfluidic model of the BBB, being able to induce different types of neuropathology (98).

Like AD, viral agents have been associated with parkinsonism disorders. These include the post-encephalitic parkinsonism linked to the 1918 influenza A H1N1 pandemic (206) and the parkinsonism associated with Epstein Barr, Coxsackie, West Nile, herpes and the human immunodeficiency (HIV) viruses (207–209). Parkinson's disease (PD) is the second most common and fastest-growing neurodegenerative disorder (210). It is characterized by dopaminergic neuronal loss in the *substantia nigra pars compacta* and the accumulation of misfolded α-synuclein (α-syn), which is found as intracytoplasmic inclusions called “Lewy bodies” (211). SARS-CoV-2 could play a role in the epidemiology of PD, since pro-inflammatory events triggered by viral infections could act as predisposing factors to the development of PD (Figure 2B steps 5 and 6) (Table 2) (212–214). This inflammatory environment can trigger the long-term neuronal loss, misfolding, aggregation, and spread of α-syn through the CNS (215, 216). Interestingly, it has been proposed that α-syn plays an essential role in response to infection, promoting a higher expression of α-syn, as occurs in the West Nile virus encephalitis (217, 218). Furthermore, α-syn aggregation can activate microglia, favoring the pro-inflammatory response and cellular damage signals, leading to slow and progressive neuronal death (219). However, it remains unknown whether SARS-CoV-2 could contribute to neurodegenerative pathogenesis, or whether it only uses the CNS as a reservoir, making it difficult for the virus to replicate, due to the low level of ACE2 receptors expressed in CNS (220).

## Conclusion

COVID-19 pandemic has become a real challenge for the scientific community around the world. Although SARS-CoV-2 mainly affects the respiratory tract, more evidence suggests that this virus can also invade the CNS causing neurological manifestations. The possible routes of SARS-CoV-2 neuroinvasion include: (1) the hematopoietic pathway *via* the BBB, (2) *via* the B-CSF, (3) *via* retrograde axonal transport through the cranial nerves, and (4) *via* the circumventricular organs. Once the virus enters the CNS, it binds to cell receptors, including ACE2. This receptor is expressed in several brain areas and in both neuronal and non-neuronal cell types. The binding of SARS-CoV-2 with ACE2 can promote neuroinflammation, hypercoagulation, microhemorrhages, BBB dysfunction, generation of reactive species, phosphorylation of tau, protein misfolding and aggregation, and neuronal death, features that are closely related to the appearance or progression of neurodegenerative diseases. Further studies on the molecular changes in the brain triggered by SARS-CoV-2 infection would facilitate timely diagnosis and therapeutic approaches.

## Author Contributions

MP-H, JL-M, and LS-R contributed to the idea formulation, reviewing of the literature, and writing and revision of the manuscript. YF-M, CH, MV-R, AL-A, PM-G, BC-C, VB-A, RA-P, CC-T, and JD-G contributed to reviewing of the literature and revision of the manuscript. All authors contributed to the article and approved the submitted version.

## Conflict of Interest

The authors declare that the research was conducted in the absence of any commercial or financial relationships that could be construed as a potential conflict of interest.
